# Trust Based Multipath QoS Routing Protocol for Mission-Critical Data Transmission in Tactical Ad-Hoc Networks

**DOI:** 10.3390/s20113330

**Published:** 2020-06-11

**Authors:** DooHo Keum, Jihun Lim, Young-Bae Ko

**Affiliations:** Department of Computer Engineering, Ajou University, Suwon 16499, Korea; dooho1000@ajou.ac.kr (D.K.); limbee94@ajou.ac.kr (J.L.)

**Keywords:** trust-based multipath QoS routing, mission-critical data, tactical ad-hoc network

## Abstract

In tactical ad-hoc networks, the importance of various tactical sensors and mission-critical data is increasing owing to their role in determining a tactical situation and ensuring the viability of soldiers. In particular, the reliability of mission-critical data has to be ensured for accurate situation determination and decision making. However, managing the network and trustworthiness in an environment where malicious nodes exist and a large amount of mission-critical data occur is a challenging issue. To solve these issues, a routing protocol is needed that can effectively detect malicious nodes and ensure the reliability and quality of service (QoS) of mission-critical data. In this paper, we propose a trust-based multipath QoS routing protocol (called MC_TQR) for tactical ad-hoc networks that can detect malicious nodes and satisfy the requirements of mission-critical data. The proposed scheme is verified using an OPNET simulator, and the results confirm the improved network performance when compared with existing schemes.

## 1. Introduction

In the future, tactical ad-hoc networks will require routing protocols to process large amounts of tactical sensor and mission-critical data securely and accurately, especially while constructing ad-hoc networks in harsh environments that have no infrastructure availability. Recently, IoT technology has been actively studied to apply to military needs such as base operations, situation awareness, healthcare, energy management, and boundary and harbor surveillance [1]. As the IoT devices increase, a networking management architecture is needed to handle the network traffic load efficiently [2]. Tactical ad-hoc networks are expected to generate large amounts of data from the various IoT sensors installed in unmanned robots, launchers, and munitions for fast and accurate command control, which emphasizes the importance of research and developments in reliable networking systems to deliver the data to the gateways. The gateway requires a technology to support essential services such as delay-sensitive applications and network management [3,4].

However, as the tactical ad-hoc networks become unmanned and the variety of devices increases, the associated cyber threats are likely to increase [5]. If a malicious node intercepts the information and consequently discards or disturbs it, mission-critical data may be lost, or the number of devices may increase, causing communication performance degradation in a resource-constrained network environment.

To solve these problems, technologies have been studied to ensure trustworthiness and QoS, taking into account trust evaluation, expected transmission count (ETX) [6], and end-to-end delay [7,8]. Moreover, bandwidth and queue management techniques have also been explored to ensure the reliability of data with high mission importance in terms of data priority.

### 1.1. Motivation

Most existing multi-path routing techniques use multi-path as an alternative path when the communication link is disconnected [9]. However, when large amounts of data are generated, transmission through a single path can be challenging to achieve by using queue and bandwidth management techniques only. In the case of low-priority data, the transfer may not be possible [10]. In this study, we attempted to guarantee the reliability and QoS by distributing and transmitting mission-critical data through multiple paths that satisfy the trustworthiness and QoS requirements of each data packet. The proposed scheme enables the effective detection of malicious attacks that might occur in tactical ad-hoc networks and ensures the reliable transfer of mission-critical data. As a result, the command and control center quickly yet accurately collects mission-critical data, which is essential for tactical operations and situational awareness.

### 1.2. Contributions

The contributions of the proposed scheme can be described as follows:

- The proposal of a trust-based multipath QoS routing algorithm for enhancing the QoS metrics in tactical ad-hoc networks.
- The trustworthiness and QoS guaranteed owing to the deployment of multipath routing algorithms to meet the mission-critical data requirements.
- The use of a flexible threshold method that considers data usage and link bandwidth for enhancing malicious node detection.

The remainder of this paper is organized as follows. In Section 2, we cover related work on trustworthiness estimation and trust-based routing for mobile ad-hoc networks and wireless sensor networks. Section 3 describes the proposed scheme in detail, and Section 4 provides a performance evaluation via comprehensive simulation studies. Finally, we conclude our work in Section 5.

## 2. Related Work

Research to ensure communication reliability between devices in various network environments has been actively conducted. The most widely used method evaluates the reliability of a specific node by observing its packet transmission behavior and can assess the energy usage, mobility, and network phase change by capturing the characteristics of each network. This section describes the existing schemes used in mobile ad-hoc networks (MANETs) and wireless sensor networks (WSNs) and introduces the trust evaluation method adopted in our proposed technique.

### 2.1. Trustworthiness Estimation

Related studies on trustworthiness estimation methods are actively being conducted, and the methods used mainly can be classified into three categories: direct trust, indirect trust, and hybrid trust.

Direct trust is a trust value calculated based on direct communication between the source node (evaluator) and its direct (immediate) neighbors and between direct nodes and its direct neighbors [11].

Indirect trust is a trust value of the evaluated node, measured or gained from indirect neighbors of the evaluator. The indirect neighbors of the evaluator are direct neighbors of the evaluated node. The indirect trust is forwarded by the direct node to the source node. When a source node receives an indirect trust value for the target node from indirect neighbors, it can calculate the trust value for the target node without performing a direct trust evaluation [11]. Another trust evaluation method, known as hybrid trust evaluation, measures the reliability of the target node based on both the trust value measured through direct trust evaluation, as well as on the indirect trust evaluation value recommended from indirect neighbors [12]. This method is useful because the accuracy of the trustworthiness may be low if the trust value is considered solely based on direct observation and may improve after the trust value evaluated by other nodes together is also considered. However, this method may result in overhead costs depending on the way both types of trust evaluations are considered and calculated.

Trust is a relative factor and can be represented as a value either confined in the interval [0, 1] or [−1,1]. The closer the trust value is to one, the more trustworthy the node, and the closer it is to −1 or zero, the less reliable the node [12].

The most commonly used method for measuring the trust value is to check whether the next-hop node has forwarded the packet after receiving the transmission from the sending node and then calculate the packet forwarding ratio (PFR) [13].

### 2.2. Trust-Based Routing

Trust-based routing is a technology that can discover and maintain routes by using trust values derived from trust evaluation and based on trust factors. In this paper, we introduce the proposed trust-based routing technology that can be utilized in MANETs and WSNs [14,15,16,17,18,19].

Among the currently used schemes, ad-hoc on-demand multipath distance vector routing (AOMDV) [14] is an extension of ad-hoc on-demand distance vector routing (AODV) [15], which is a typical reactive routing protocol in MANETs. AOMDV is used for multipath routing because it is a search feature for linking disjoint multiple paths during path discovery.

Ad-hoc on-demand trusted-path distance vector routing (AOTDV) [16] is a trust-based multipath routing protocol that extends AOMDV. Therefore, although the basic routing path discovery method of AOTDV is similar to that of AOMDV, the process in which the source node transmits the RREQ (route request) packet and the destination node that receives it transmits the RREP (route reply) packet is different. AOTDV considers both the hop count and the path trust value (*PTV*)as routing metrics. To calculate the *PTV*, it is necessary to evaluate the reliability of each node. In AOTDV, the ratio of the nodes that forward the target packet per unit time using the direct trust evaluation method is measured by considering both the control and the data packets. Based on this ratio, the *PTV* is accumulated by multiplying the trust values of all the nodes on the transmission path of the RREP packet, and each node updates the *PTV* in the routing table. After the trust path is created, the source node selects a path that satisfies the trust value required to transmit the important data packet and transmits the data. However, this can cause problems such as bottlenecks because it selects one path when transmitting data.

Trust-based QoS routing (TQR) [17] introduced the concept of trust and QoS metric estimation. The authors demonstrated the performance of trust values and of discovering and maintaining the most reliable paths by properly considering trustworthiness and QoS metrics. They measured the ETX, propagation delay, and transmission delay for calculating the QoS metric and calculated them with trust values. However, in the case of a bottleneck, the delay accumulated in the queue was not considered, and only a single path was used to transmit the data, making reliable communication difficult. This problem can be solved by creating an algorithm that periodically checks the trust value of the trusted multipath and efficiently transmits mission-critical data simultaneously over these paths.

Centralized trust-based efficient routing with authentication (CENTERA) [18] introduced a gateway-assisted trust evaluation technique. CENTERA uses a base station (BS), which can effectively collect trust information from all the nodes in the topology, and calculates the best possible routes after detecting and isolating the malicious nodes. The BS creates a global view of the network topology and evaluates the trust value of each node by calculating three metrics: maliciousness, cooperativeness, and competency. The BS can detect malicious node types, such as those sending false or illogical information, those not reliably forwarding the packets from other nodes, or those unable to deliver the packets to the BS correctly. The malicious nodes are then isolated for a certain duration based on their history. The BS increases the level of bad reports or protective observation for all the nodes showing bad behavior while reducing the level of harm to well-behaving nodes. The BS then periodically distributes updated behavior-related information to all the nodes using an efficient method.

However, CENTERA uses only the PFR to perform the trust evaluation of the nodes, therefore rendering the nodes vulnerable to denial of service (DoS) attacks that diminish the resources of the system and prevent its intended operations. In this study, we were able to solve this problem by considering the QoS factors in addition to the PFR.

Recently, the work in [19] presented a QoS-aware trust-based routing protocol, named “SQEER” (secured quality of service-aware energy efficient routing). In SQEER, multiple routing metrics such as the path-trust value and residual energy are utilized to figure out which path can meet the required level of trustworthiness and QoS of energy-hungry sensor networks. However, it has limitations in ensuring reliability for mission-critical data transmission. Although it calculates trust values for multiple paths, only one path is used for data transmission, and no consideration is given to other data of high importance.

Compared with the existing works, our trust-based multipath QoS routing protocol is novel in two ways. First, it fulfills the performance requirements by taking into account tactical factors to transmit mission-critical data. Second, a path selection process is proposed to satisfy the performance of reliability and QoS required in the tactical environment.

## 3. Proposed Scheme

This section introduces our trust-based QoS multipath routing scheme for the secure and reliable transmission of mission-critical data. The proposed MC_TQR is based on the AOMDV multipath discovery and maintenance process and can detect malicious nodes and guarantee the reliability of mission-critical data. This section describes the mission-critical data characteristics, the path discovery, and maintenance techniques of the proposed scheme.

### 3.1. Performance Requirements For Mission-Critical Data Delivery

The U.S. Army Unified Capabilities (UC) Reference Architecture (RA) report provides resource assurances and a service differentiation between real-time and non-real-time mission-critical data over the network [20]. UC services provide support to all operational phases and facilitate the convergence of the operating and generating forces. UC services facilitate more timely delivery of emerging UC technologies and provide increased mission effectiveness.

Army UC services are required to be delivered in accordance with different priority/precedence levels with connectivity [20]. Accordingly, voice, video, audio, and data for multimedia conferencing are delivered over the networks using multi-level precedence and preemption (MLPP). MLPP-based services are also known as precedence-based assured services (PBAS), with five priority levels from the lowest to the highest [21]: ROUTINE (R), PRIORITY (P), IMMEDIATE (I), FLASH (F), and FLASH OVERRIDE(FO), which can be mapped to future combat system (FCS) attributes. FCS is a joint networked system of systems and is connected using an advanced network architecture that facilitates situational awareness, joint connectivity, and synchronized operations [22]. The system operates as a system of systems that networks the existing systems and has the capacity to add yet to be developed systems also to meet the future requirements of the Army’s FCS brigade combat teams. The FCS data traffic includes information regarding collaboration command control (C2), situational awareness, target data, fire requests, medical states, sensor tasking data, and terrain data. In addition, the system can classify the per-hop behavior (PHB) into expedited forwarding (EF), assured forwarding (AF), and best effort (BE) according to the transmission priority and can map it according to the required FCS traffic attributes. Table 1 summarizes the performance requirements of the various FCS traffic data elements, UC applications, and QoS mapping factors based on the priority/precedence levels referred to in [21]. In the present study, the elements were applied to an algorithm by considering the mapping relationships among the priority-based traffic attributes.

### 3.2. Trustworthiness and QoS-Based Path Discovery and Selection Procedure

The path discovery method of the proposed scheme works similarly to the AOMDV, which is a multipath routing method used in existing ad-hoc networks. However, there are differences in the methodologies used to discover and maintain the trusted paths. In the initial step of the process, the gateway node sends an RREQ message to each sensor node, which when received by the source node responds with multiple RREP messages to discover a trusted route. While searching for a trusted path, each node observes the behavior of its neighboring nodes to obtain their node trust value (NTV), which is obtained by observing their behavior through the promiscuous mode and is calculated using the commonly used PFR [16]. The PFR is determined by checking whether a receiving node forwards the packet and is then used as an index to detect malicious behavior, such as any malicious node arbitrarily discarding or not transmitting the received packet. To check whether normal packet forwarding is being performed, all data transmission is monitored, and the trustworthiness is calculated periodically against a set unit time. The PFR is calculated by using Equation (1) below, based on the number of packets transmitted by a node i to node jduring the set unit time and the number of packets forwarded by the node j after receiving them from i.

(1) $$PFR_{i,j}^{d}\left(t\right)=\frac{F_{i,j}\left(t\right)}{S_{i,j}\left(t\right)}$$

$S_{i,j}\left(t\right)$ denotes the total number of packets transmitted by node i to j during the unit time *t*, and $F_{i,j}\left(t\right)$ denotes the number of packets forwarded by node j after receipt from i during the unit time *t*.

$PFR_{i,j}^{r}\left(t\right)$ denotes the trust value for node j that was recommended to node i by the neighboring node. $PFR_{i,j}^{r}\left(t\right)$ can be calculated based on the trust values of k neighbor nodes, as given in Equation (2).

(2) $$PFR_{i,j}^{r}\left(t\right)=\frac{1}{n}\sum_{k=1}^{n}PFR_{k,j}^{d}\left(t\right)$$

Using the above formula, the transmitting node i can calculate the NTV for the receiving node j through the following Equation (3). $w_{1}$ and $w_{2}\left(w_{1},w_{2}\geq 0,w_{1}+w_{2}=1\right)$ were assigned as weight factors for $PFR_{i,j}^{d}\left(t\right)$ and $PFR_{i,j}^{r}\left(t\right)$, respectively.

(3) $$NTV_{i,j}\left(t\right)=\left(w_{1}\times PFR_{i,j}^{d}\left(t\right)\right)+\left(w_{2}\times PFR_{i,j}^{r}\left(t\right)\right)$$

In this study, we added the previous node ID information to the packet header to confirm whether the packet transmitted by node j had been received from node i only or from any other neighbor node. If a node exhibits malicious behavior that involves any arbitrary discarding of packets, such as a black hole or gray hole attack, the NTV for the node decreases. Thus, a node can be labeled as malicious if its NTV falls below a threshold value. The collected NTV values of all the nodes derived through the above method are then used to calculate the PTV in the trust path discovery process.

Table 2 shows the meaning of the different node trust levels [16]. Before any interaction between the nodes, the initial trust value is 0.75 (less trustworthy node). A threshold value (γ) is assigned (considered as the blacklist threshold) and is used to pinpoint the malicious nodes. It can be set differently depending on the user and operator intentions. The threshold reflects the communication environment of the node and is flexibly calculated as shown in Equation (4).

(4) $$\gamma =\frac{Currentbandwidth}{Linkbandwidth}\times \mu \;\left(0\leq \gamma <0.75\right)$$

The reason for this calculation is to determine clearly whether the communication status is bad or an attack by a malicious node. The variable μ is a constant (1>μ≥0). It can be set differently depending on the user and operator intentions. When the path bandwidth usage is low, the flexible threshold values are also reduced to allow the detection of malicious nodes with low packet drop attacks. If the path bandwidth usage is high, it is difficult to ascertain whether the network performance degradation is due to a bottleneck or a malicious node, and therefore, the flexible threshold is increased to help in the careful determination of path exclusions.

We set the minimum NTV of the nodes in the path equal to the *PTV*, as shown in Equation (5) [23].

(5) PTV=minNTV(0≤PTV≤1)

In tactical ad-hoc networks, mission-critical data have different requirements in terms of urgency and importance. Generally, the more mission-critical the data are, the more secure and trusted the required paths. Table 3 shows an example of the trust requirements of the data packets [16]. They can also be set differently depending on the user and operator requirements.

In case of a delay, the network delays measured by each sensor node are accumulated and summed up at the gateway node. To prevent indiscriminate path generation, the sensor node receiving the plural of RREQs generates RREPs for up to three valid paths after considering the number of reliability levels of the data packets. The gateway node receiving the RREP updates k paths. The gateway node periodically checks the *PTV*, ETX, and E2Edelay ($\sigma_{p}\left(s\right)$) values to calculate the path QoS and trust value (PQTV), as shown in Equation (6), and sends them to the sensor nodes. The sensor nodes can check the trusted paths based on the received value.

(6) $$PQTV_{p}\left(t\right)=\sigma_{p}\left(s\right)\times ETX_{p}\times \left(1-PTV\right)$$

In general, multipath routing in traditional ad-hoc networks can be divided into two types. The first simultaneously transmits the packets over multiple paths to guarantee the transmission reliability of the data packet. The second primarily uses the best path from among multiple paths and then uses alternative paths if the link quality degrades owing to a bottleneck or a link disconnection. Our scheme proposes a multipath selection for mission-critical data to solve the problems of bottlenecks and degradation of link quality and to enable the simultaneous transfer of more important data over the trusted path.

Trust path selection is based on the trust value of each path and the priority of mission-critical data. Transmission of data with high mission criticality requires the selection of a more reliable path.

**Figure 1:**
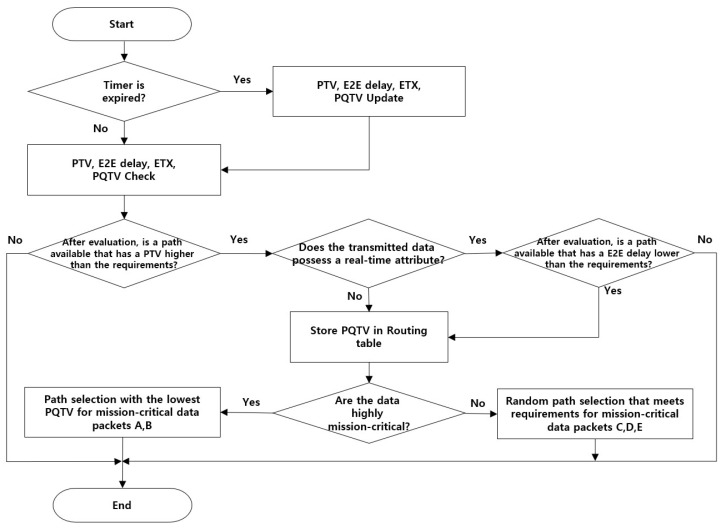
Overall process for selecting paths that satisfy the trust requirements. ETX, expected transmission count.

Figure 1 shows the overall process of path selection to satisfy the requirements of the proposed scheme. Here, the sensor node periodically updates and checks the trust value received. The requirements of mission-critical data are compared to the updated *PTV*s and E2E delay, and the *PQTV* of the valid paths are stored in the routing table. Finally, the sensor node can identify the best priority path over which mission-critical data should be transmitted and adaptively selects the path with the optimal trustworthiness and QoS.

We compared the *PTV* requirements of each data packet with the actual measured *PTV* to determine the reliability of the path for transmitting mission-critical data. Because mission-critical data contain important information that should not be exposed to malicious nodes, they can be transmitted only when the trust value for the path meets the requirements.

If the *PTV* is satisfactory, the delay time of the real-time data being sent is checked and compared with the required delay for the data. Mission-critical data transmission requirements are defined based on communication status, priority/precedence, etc. If there is no path that satisfies the requirements, mission-critical data such as voice, which is real-time and important, are not transmitted through the network until a trusted path is guaranteed. Therefore, in this case, the operator should communicate using alternative methods, such as by using a separate voice communication frequency.

**Figure 2:**
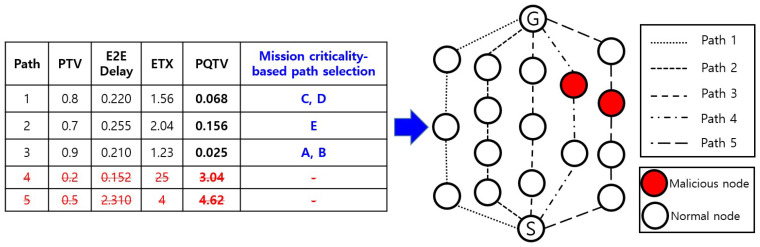
Example of path selection based on mission-critical data priority.

For real-time data packets that satisfy the required performance parameters, the *PQTV* in the routing table is updated; for unsatisfactory performance, the packet is neither stored in the routing table nor transmitted. After successful fulfillment of the performance requirements, highly critical mission data are transmitted through the path with the minimum value among the stored *PQTV*s. Relatively low-critical mission data are randomly selected from paths that satisfy the requirements. In this study, it was assumed that the data packets A and B had high mission criticality, and the packets C, D and E had low priority, but the system could be operated according to the intention and preferences of the operator. Figure 2 shows an example of the methodology employed for assigning a path for transmission of mission-critical data from the sensor node to the gateway node.

The *PQTV* evaluation in our experiment resulted in values of 0.068, 0.156, 0.025, 3.04, and 4.62 for Routes 1, 2, 3, 4, and 5, respectively. Therefore, transmission of the mission-critical data packet A was assigned to Route 3, which satisfied all the requirements. In the case of B, Routes 1 and 3 both satisfied all the requirements. For transmitting B, the node selected Route 3 with the lowest *PQTV* value. In the case of the C, D, and E data packets, the paths satisfying the requirements were 1, 2, and 3, which were randomly selected and used after being assigned, as shown in Figure 2. As shown in Paths 4 and 5, if a malicious node performed a gray hole or DoS attack, it was detected, and the path was excluded. Each sensor node could transmit data along a path that guaranteed trustworthiness and QoS because it detected and excluded malicious nodes beforehand.

### 3.3. Maintenance of Trustworthy and QoS-Assured Paths

Path maintenance is a mechanism to decide how to use or search alternative paths if network conditions change (e.g., the occurrence of link bottlenecks due to attacks by malicious nodes or large amounts of data). If the mechanism discovers a node that is unable to act normally, it sends a route error message (RERR) with a blacklist to the gateway node. When the gateway node receives information that the path has been compromised, it can try to use another known path to the sensor node or can perform a path discovery routine again to search for a new path.

Path maintenance verifies the path validity at specific time intervals. When the path cache entry exceeds the maximum validity time, a new path discovery procedure is also initiated. In addition, control messages are sent and received periodically for the selection and maintenance of trustworthy paths with satisfactory QoS. The gateway node calculates the *PQTV* based on the values received from nodes on valid paths and sends it to each sensor node. The sensor node updates the *PTV*, E2E delay, ETX, and *PQTV* received from the gateway. As a result, the gateways and sensor nodes can periodically check the trustworthiness and QoS values for each path. Consequently, a new optimal path is established after the completion of this procedure.

## 4. Performance Evaluation

In this section, we describe the settings of the simulation environment, as shown in Table 4, that we used for verifying and evaluating the performance of the proposed technique. We used OPNET 18.0 as the network simulator and implemented our proposed protocol (MC_TQR) and compared it with the routing protocols AOMDV, AOTDV, and TQR. The simulation was performed with 50 nodes in a partial mesh topology that was placed in a 1000 m × 1000 m area. There were 49 sensor nodes and a fixed gateway node. The ratio of malicious nodes was set to 0–40%, and two types of attacks (gray hole and DoS attacks) were performed. In gray hole attacks, malicious nodes could randomly drop data packets, with a dropping ratio in the range of 0.4–0.8 [17]. In a DoS attack, malicious nodes could periodically send a certain amount of packets to the target node. Data information was fixed according to mission criticality by using the constant bitrate (CBR) traffic model by taking into account the type, size, and period of audio and video data transmissions in a tactical network environment [24,25]. In addition, our experimental environment generated more data than the link bandwidth capacity of the path to mimic the tactical ad-hoc network conditions. The media access control (MAC) protocol included carrier sense multiple access with collision avoidance (CSMA/CA), and the physical layer (PHY) was set to 2 Mbps (operated in the soldier radio waveform) to imitate the resource-constrained ad-hoc communication in the military environment [26]. On the performance evaluation scale, the packet delivery ratio (PDR), end-to-end delay, and throughput were compared and analyzed. The PDR was calculated by considering the number of packets sent and received from the source node to the destination node, and the delay measured the end-to-end delay time from the time the packet was sent by the transmitting node to the time the packet was received by the destination node. The throughput was calculated by measuring the number of packets transmitted within a specified time.

**Figure 3:**
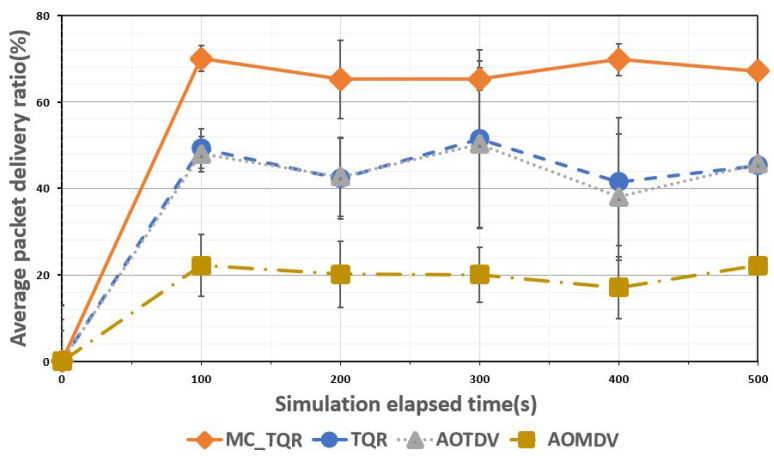
Average packet delivery ratio over elapsed time.

**Figure 4:**
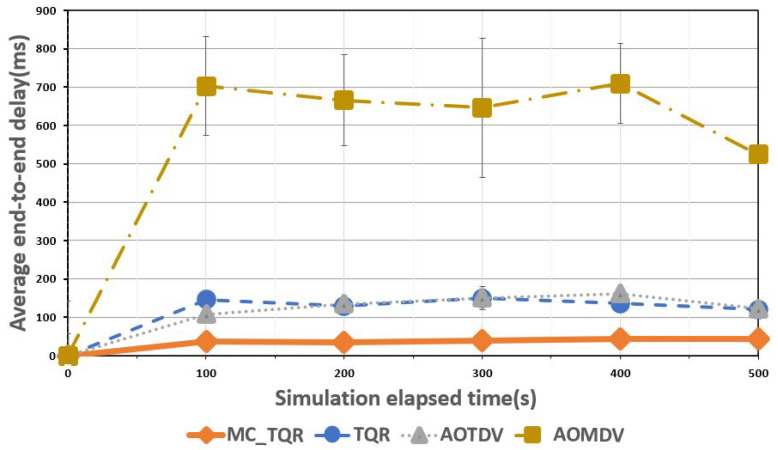
Average end-to-end delay over elapsed time.

**Figure 5:**
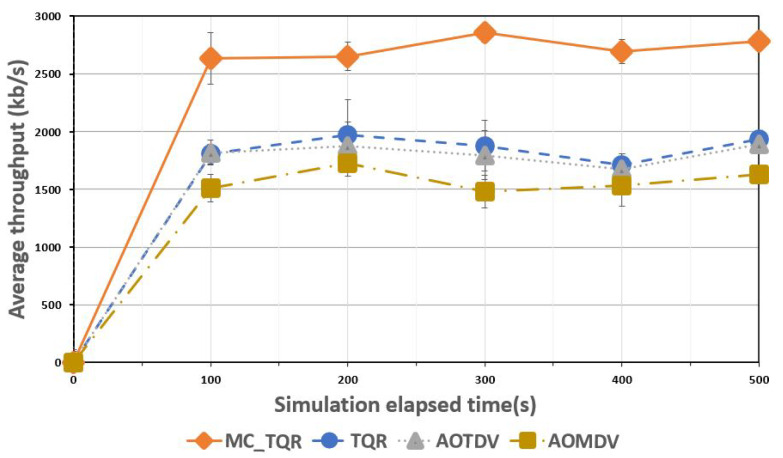
Average throughput over elapsed time.

Figure 3, Figure 4 and Figure 5 show the performance evaluation results when the malicious node percentage was 30%. Figure 3 shows the average rate of packet delivery over the elapsed time. AOMDV reported the lowest PDR results compared to the other schemes because the gray hole and DoS attacks could not be detected using its routing metric, which considered only the hop count, where the data were transmitted over the shortest hop. AOTDV and TQR selected a trusted path by using routing metrics that considered reliability, and therefore, they could transmit the data over a reliable path by detecting the malicious nodes. However, owing to the high ratio of malicious nodes (30%) and occurrence of bottlenecks because of data transmission through a single path, their average PDR result was less than 50%. The proposed MC_TQR scheme selected the trusted paths using routing metrics that considered both reliability and QoS. Load balancing was guaranteed because of the detection of malicious nodes and the distribution of data through trusted multiple paths. As a result, even though the malicious node ratio was 30%, our protocol showed a PDR performance of approximately 70%, which was the highest among all the schemes.

Figure 4 shows the average end-to-end delay over the elapsed time. In the case of AOMDV, the route with the smallest number of hops was selected, and the data were transmitted using the hop count metric. As only the shortest hop path was considered, AOMDV did not detect the nodes performing the attacks and transmitted the data on the paths with such nodes, resulting in the highest end-to-end delay when the malicious node ratio was 30%. This was because the delay in the packet processing time at the intermediate nodes accumulated continuously owing to the DoS attack, resulting in an end-to-end delay of approximately 600 ms, which could result in a disastrous tactical network situation. Conversely, AOTDV and TQR periodically updated the most reliable path to transmit the data. TQR additionally used metrics to factor in the ETX and delay to update the trusted path faster than AOTDV, resulting in a lower end-to-end delay. However, because the data packets were transmitted over a single path, this resulted in an end-to-end delay of approximately 150 ms. MC_TQR showed the lowest end-to-end delay results compared with the other schemes because it detected the malicious nodes and then selected multipath to ensure load balancing.

Figure 5 shows the average throughput over the elapsed time. The throughput was determined by calculating the data transmission in terms of packet size, generation rate, and overhead (ACK, back-off, etc.) for a given unit time. In this experiment, various tactical data features were used, as shown in Table 4. MC_TQR, which used metrics that considered both reliability and QoS to search for a trusted path quickly and also guarantee load balancing, reported a throughput of approximately 2800 kb/s. In tactical ad-hoc networks, mission-critical data should be transmitted quickly and accurately, and the effectiveness of the proposed technique was confirmed by the throughput results, when compared with other schemes. TQR and AOTDV reported similar throughput results of approximately 1900 kb/s, with TQR showing a slightly superior throughput because of its use of metrics such as trustworthiness and QoS. AOMDV reported the lowest throughput (approximately 1600 kb/s) owing to its technique of transmitting data without updating the path, while remaining exposed to DoS and gray hole attacks. In tactical ad-hoc networks, such mission-critical data could not be processed effectively, which could cause major problems.

**Figure 6:**
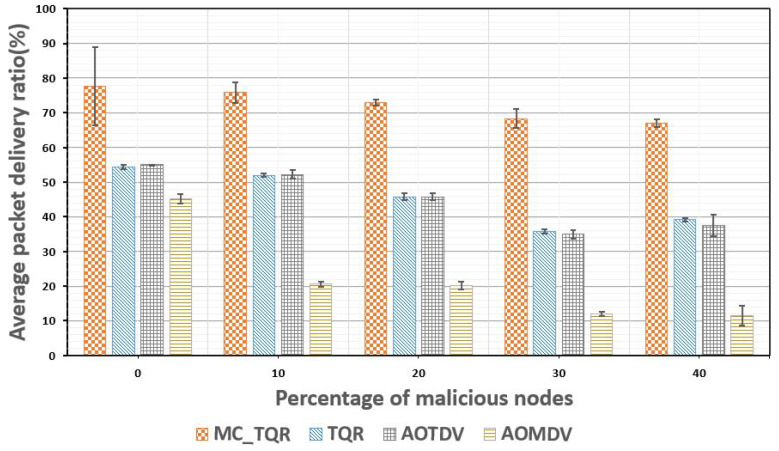
Average packet delivery ratio plotted against the percentage of malicious nodes.

Figure 6 presents the PDR results based on the percentage of existing malicious nodes. In this experiment, we compared and analyzed the PDR performance while increasing the proportion of malicious nodes in the network topology from 0 to 40%. The average PDR was measured by considering a base tactical network environment where there was a delay due to a bottleneck because of a higher data usage than the link bandwidth even in the absence of any malicious node. Overall, as the percentage of malicious nodes increased, the PDR decreased owing to the increased processing time required for performing and analyzing the trust evaluations. AOMDV displayed significantly lower PDR results as the percentage of malicious nodes increased. TQR and AOTDV showed similar reductions in the PDR results as the percentage of malicious nodes increased. The reason was that both schemes used similar trust-based metrics, so the data could be transmitted by selecting the same path according to the topology network. MC_TQR used an algorithm that additionally distributed and transmitted the data while using the trust and QoS metrics and, therefore, demonstrated the capacity to effectively transfer a larger amount of data than other schemes.

**Figure 7:**
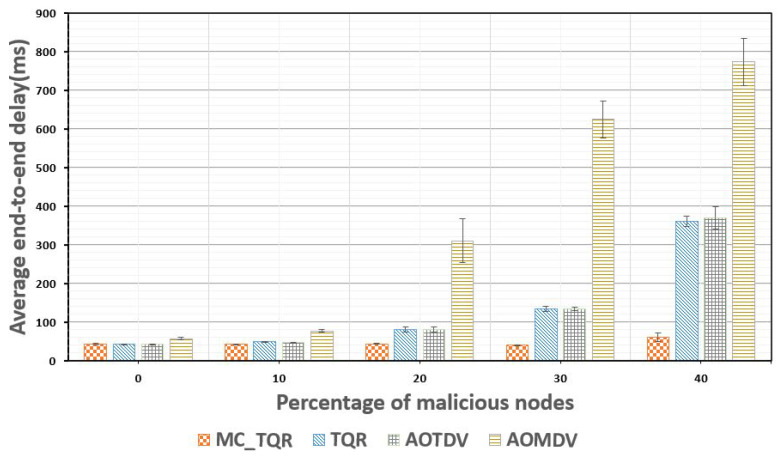
Average end-to-end delay plotted against the percentage of malicious nodes.

Figure 7 shows the average end-to-end delay based on the percentage of malicious nodes. As the percentage of malicious nodes increased, the average end-to-end delay increased for all the schemes. Considering a tactical ad-hoc network environment, even for no malicious nodes, the system may have a 40–60 ms delay because it set the data usage higher than the link bandwidth. The results of the proposed MC_TQR protocol demonstrated that the delay did not exceed 60 ms even when the percentage of malicious nodes increased to 40%. Therefore, the reliability of the scheme as a reliable routing protocol was proven owing to its observed end-to-end delay being much lower than the 220 to 1000 ms delay required in tactical ad-hoc networks. On the other hand, the results for TQR and AOTDV indicated that they did not satisfy the required delay conditions as the percentage of malicious nodes increased. In the case of TQR, which used a routing protocol that considered both reliability and QoS, a delay still occurred when only one path was used for transmission. The AOMDV protocol continued transmitting data through a path even after it was exposed to a DoS attack, and the end-to-end delay increased significantly as the percentage of malicious nodes increased.

**Figure 8:**
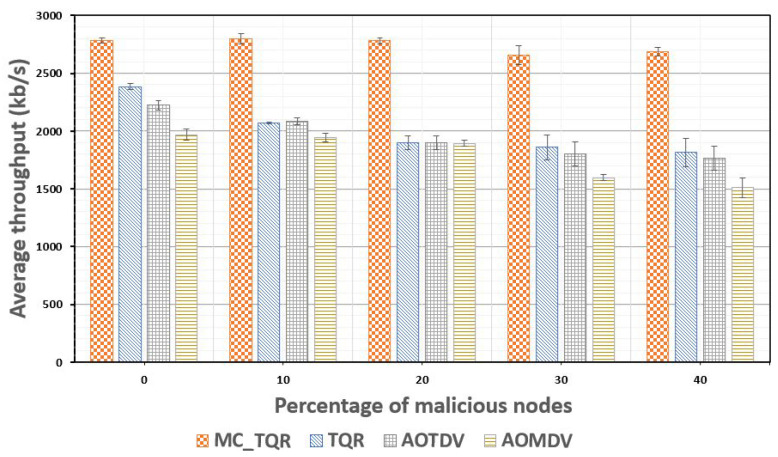
Average throughput values plotted against the percentage of malicious nodes.

Figure 8 shows the average throughput based on the percentage of malicious nodes. As the percentage of malicious nodes increased, the throughput decreased in all schemes. TQR, AOTDV, and AOMDV reported a throughput of approximately 1500–2000 kb/s and displayed a significant decrease when the percentage of malicious nodes increased. This anomaly made it difficult to achieve reliable communication in tactical wireless networks, which require guaranteed reliability and transmission urgency of mission-critical data. The proposed MC_TQR protocol reported a processing throughput of approximately 2800 kb/s even when the malicious node percentage increased to 40%, which verified that it could stably process mission-critical data.

## 5. Conclusions

This paper proposed a trust-based multipath QoS routing technique that detected malicious nodes in a tactical network and transmitted mission-critical data through paths that guaranteed reliability and quality of service. As the transmission of tactical data must be guaranteed owing to their urgency and reliability attributes, research to ensure the reliability and quality of service is essential. Therefore, the performance requirements were applied to the algorithm in consideration of the mapping relationship between priority-based tactical traffic attributes. The technique proposed in this paper could help transmit the data quickly and safely while satisfying these requirements. From the experiments conducted, it is noted that our proposed scheme showed better performance in terms of PDR, end-to-end delay, and throughput when compared to related schemes. Future works on this proposed scheme will apply machine learning to improve reliability.

## Figures and Tables

**Table 1:** Future combat system (FCS) traffic, Unified Capabilities (UC) applications, and QoS mapping based on priority/precedence levels. PHB, per-hop behavior; C2, command control; FO, FLASH OVERRIDE; EF, expedited forwarding; F, FLASH; I, IMMEDIATE; P, PRIORITY; R, ROUTINE; AF, assured forwarding; BE, best effort.

Mission Critical Level	FCS Traffic	Data Type	E2EDelay Requirement	Time Attribute	Priority/Precedence	PHB
A	Collaborate C2	Voice	220 ms	Real-time	FO	EF
	Fire request					
	Medical status					
B	Collaborate C2	Video	220 ms	Real-time	FO, F, I, P, R	AF4
	Situation awareness					
C	Situation awareness	Chat	300 ms	Non-Real	FO, F, I, P, R	AF3
D	Damage assessment	Short messaging, sensor data	1000 ms	Non-Real	FO, F, I, P, R	AF2
	Sensor tasking					
E	Terrain data	Bulk data	300 ms	Non-Real	Not Applicable	BE

**Table 2:** Different node trust levels with their meanings. γ denotes the threshold value. NTV, node trust value.

Level	NTV	Meaning
1	[0.9, 1]	Trustworthy node
2	[0.75, 0.9]	Less trustworthy node
3	[γ, 0.75]	Suspect node
4	[0, γ]	Malicious node

**Table 3:** Examples of the trust requirements for different data packets. PTV, path trust value.

Level	PTV	Meaning
1	[0.9, 1]	Extremely important data
2	[0.75, 0.9]	Important data
3	[0.65, 0.75]	Less important data

**Table 4:** Simulation environment settings. AOMDV, ad-hoc on-demand multipath distance vector routing; AOTDV, ad-hoc on-demand trusted-path distance vector routing.

Parameters	Values
Simulator	OPNET 18.0
Simulation time	500 s
Routing Protocols	AOMDV, AOTDV, TQR, MC-TQR
Number of nodes	50
Percentage of malicious nodes	0–40%
Traffic type	VoIP G.723.1 (24 bytes)
	Video surveillance H.264 (500 bytes)
	Lighting sensor, chat (100 bytes)
	Fire alarm, health sensor, message (120 bytes)
	CCTV camera, bulk data (2000 bytes)
MAC	CSMA/CA
PHY	802.11b (2Mbps)
μ	0.5
$w_{1}$	0.5
$w_{2}$	0.5
